# Comparative Analysis of Cell-Free DNA Fragmentation Patterns in Canines with Sarcoma and Tumor-Free Canines and Humans

**DOI:** 10.1158/2767-9764.CRC-25-0373

**Published:** 2026-02-13

**Authors:** Patricia Filippsen Favaro, Yinghua Wang, Bradon R. McDonald, Tammy Xiong Wang, Samanyu Jadhav, Han-Yun Hannah Cheng, Clayton T. Marcinak, Xuan Pan, Muhammed Murtaza

**Affiliations:** 1 Center for Precision Medicine, University of Wisconsin-Madison, Madison, Wisconsin.; 2 Department of Surgery, University of Wisconsin-Madison, Madison, Wisconsin.; 3 Carbone Cancer Center, University of Wisconsin-Madison, Madison, Wisconsin.; 4 Department of Medical Sciences, School of Veterinary Medicine, University of Wisconsin-Madison, Madison, Wisconsin.; 5 Wisconsin Blood Cancer Research Institute, Madison, Wisconsin.

## Abstract

Plasma cell-free DNA (cfDNA) analysis holds potential to improve the detection of naturally occurring cancer in dogs, which can also serve as important model organisms for human cancer. However, there are limited comparative data on the characteristics of canine and human cfDNA. We characterized cfDNA fragmentation in 254 plasma samples from 54 healthy dogs and 54 dogs with naturally occurring sarcomas and compared them with 35 samples of human cfDNA. Using electrophoresis, whole-genome sequencing with both short and long reads, and multiplexed quantitative PCR, we assessed both fragment size distribution and fragment end sequences. We then trained a random forest classifier to distinguish healthy dogs from those with sarcomas based on cfDNA fragmentation features. Canine cfDNA fragment size distributions showed a striking deviation from humans, including a median of only 39% of fragments between 50 and 700 base pairs versus 84% in human samples. Fragment end nucleotides were more random in dogs than in humans at multiple size ranges. Similar to human patients with cancer, dogs with sarcomas had detectable copy-number changes and characteristically shorter cfDNA fragments relative to healthy dogs, enabling classification of cancer samples with 91% accuracy. Our results demonstrate key differences in cfDNA fragmentation between dogs and humans and highlight the potential for comparative translational research to advance blood-based early cancer detection in both species.

**Significance:**

cfDNA analysis in dogs with naturally occurring cancers can enable precise detection of canine cancer and serve as a model system for human oncology. In this study, we observed differences in cfDNA fragmentation patterns between humans, healthy dogs, and dogs with sarcomas. Our results highlight the opportunity for comparative oncology research, the development of canine cfDNA diagnostics, and the investigation of biological mechanisms driving differences between humans and dogs.

## Introduction

Naturally occurring cancer is the cause of death for approximately 45% of dogs over the age of 10 years. Given this high incidence and shared environmental exposures, comparative analysis of canine cancer can serve as an informative model system for human oncology (1, 2). Most canine cancers are diagnosed at an advanced stage after clinical signs appear, often with local invasion or metastasis, contributing to a poor clinical prognosis (3, 4). Early cancer detection has the potential to significantly improve treatment efficacy and prognosis (5), and longitudinal studies of canine cancer would benefit from inexpensive, noninvasive diagnostic tools. However, there are no widely accepted methods for early detection or treatment monitoring of cancer in dogs beyond imaging, which lacks the necessary sensitivity (6, 7).

In humans, many studies have demonstrated the potential of analyzing cell-free DNA (cfDNA) in plasma for early cancer detection (8, 9), monitoring therapeutic response (10), and detecting minimal residual disease (11). Human plasma cfDNA displays highly reproducible fragment length distributions, fragment end motifs, and nonrandom fragmentation patterns across genomic regions (12). Importantly, tumor-derived cfDNA fragments vary in all of these characteristics (13, 14), and these differences can be leveraged to improve cancer detection through the enrichment of shorter fragments and genome-wide fragmentation analysis (15–17).

In comparison with these advances in human medicine, characterization of canine plasma cfDNA fragmentation is limited so far (18–20). It remains unknown whether cfDNA concentration and fragmentation patterns in dogs resemble those observed in humans and how these characteristics differ in dogs with cancer. Here, we use a combination of fluorometry, automated electrophoresis, short- and long-read whole-genome sequencing, as well as a custom multiplexed quantitative PCR (qPCR) assay to analyze cfDNA from 54 healthy dogs and compare its characteristics with those found in humans. We then investigate how these features differ between healthy dogs and 200 samples taken from 54 dogs diagnosed with sarcomas.

## Materials and Methods

### Canine patient enrollment

Canine patients presenting between December 2020 and June 2023 at the University of Wisconsin Veterinary Care were enrolled under protocol number V006456-A01 approved by the Institutional Animal Care and Use Committee, and informed consent was obtained from all owners. Healthy dogs were enrolled as part of their participation in the Vaccination Against Canine Cancer Study. Based on conventional histologic and cytologic evaluation, dogs diagnosed with osteosarcoma, hemangiosarcoma, or soft-tissue sarcoma were enrolled in the study based on convenience sampling. Dogs were excluded if they had severe infectious or inflammatory disease, severe trauma, or known cancers other than sarcomas. Toy and small breed dogs were also excluded due to the difficulty of collecting sufficient blood volume.

### Canine sample collection and analysis

Blood samples were collected from the jugular or cephalic vein from 54 dogs with cancer and 55 age-matched healthy dogs clinically screened for cancer. The collection tube was determined by dog size: Streck cfDNA blood tubes for dogs in which the veterinarian determined that at least 7 mL of blood could be collected and K2-EDTA blood tubes of 3 or 6 mL capacity for smaller dogs. Streck tubes were processed within 24 hours of collection, and K2-EDTA tubes were processed within 2 hours. Blood samples were spun at room temperature for 10 minutes at 1,600 × *g* for Streck tubes and 820 × *g* for K2-EDTA tubes. Blood processing protocols are available online for K2-EDTA tubes (https://dx.doi.org/10.17504/protocols.io.ewov11bzyvr2/v1) and Streck tubes (https://dx.doi.org/10.17504/protocols.io.x54v9541ml3e/v1). The supernatant was distributed in 1 mL aliquots and spun for 10 minutes at maximum speed (16,000–20,000 × *g*) and stored at −80°C. The buffy coat was collected and stored at −80°C. All dogs had their clinical information (Supplementary Table S2) collected using REDCap (21). cfDNA was extracted from 2 to 3 mL of plasma using the MagMAX cfDNA kit (Applied Biosystems) on the KingFisher instrument with a final elution volume between 15 and 30 μL of elution buffer. The automated protocol and additional steps are available online (https://dx.doi.org/10.5281/zenodo.17555907). Electrophoresis was performed using the 4200 TapeStation System (Agilent) with cfDNA ScreenTapes according to the manufacturer’s instructions. cfDNA quantification was performed using Qubit 4 (Invitrogen).

### Human cfDNA collection and analysis

Plasma from healthy human volunteers was used to compare canine and human cfDNA. Enrollment, blood collection, and plasma separation were performed by the University of Wisconsin (UW)-Madison Translational Science BioCore BioBank with written informed consent from the volunteers, under ethical guidelines approved by the Institutional Review Board of the University of Wisconsin-Madison, in accordance with the Belmont Report (IRB protocol 2016-0934). Blood samples were collected in K2-EDTA tubes and spun down with two rounds of centrifugation within 1 hour of sample collection (using the same protocol as EDTA tubes from dogs), and plasma was stored at −80°C. cfDNA was extracted from 1 mL of plasma using the MagMAX cfDNA kit (Applied Biosystems) on the KingFisher instrument and eluted in 30 μL of elution buffer. Electrophoresis was performed using 4200 TapeStation System (Agilent) with cfDNA ScreenTapes according to the manufacturer’s instructions.

### Canine cfDNA qPCR

To assess concentration and fragment size, we designed a multiplexed qPCR assay to detect short and long fragments of canine cfDNA. Both primer/probe sets detect repetitive regions of approximately 99 bp (short fragments, *LINE99*) and 598 bp (long fragments, *PECAM1*), respectively (Supplementary Table S2). The *LINE99* assay was previously described (22), whereas we designed the *PECAM1* (ENSCAFG00000011740) assay for the current study. To calculate a standard curve for absolute quantification and the calculation of the fraction of short DNA fragments, we prepared a dilution series with genomic DNA from commercially available beagle peripheral blood cells (Biochain). In each reaction, we used 0.4 μmol/L of each primer and 0.2 μmol/L of each probe. qPCRs were run at a final volume of 10 μL using the TaqMan Fast Advanced Master Mix (Applied Biosystems). Plates were cycled as follows: a single cycle of 50°C for 2 minutes, a single cycle of 95°C for 20 seconds, and 40 cycles of 95°C for 1 second, 56°C for 1 second, and 60°C for 20 seconds. Signal was captured during the 60°C step. The fraction of short cfDNA was estimated using the DNA quantification of both assays:

$$short\;index\;=\;\frac{\left(LINE\;-\;PECAM\right)}{LINE}$$

### Human cfDNA digital PCR

A digital PCR assay was used to conduct an equivalent analysis of human cfDNA, described previously (23). Briefly, the assay consists of five primer pairs targeting single-copy housekeeping genes that amplify all fragments to generate average amplicon lengths of 71 bp. An additional set of four primer pairs are included that only amplify longer fragments and generate average amplicon lengths of 471 bp. Based on the quantity of DNA amplified in each size range, we calculate the fraction of short fragments using a method similar to the one used for canine cfDNA quantification.

### Canine and human whole-genome sequencing

Plasma cfDNA whole-genome sequencing for dogs was performed using the Illumina NextSeq 2000 and Oxford Nanopore PromethION platforms. Short-read sequencing libraries were prepared with an average cfDNA input of 1.2 ng, using the ThruPLEX DNA-Seq HV library prep (Takara) with 10 to 15 amplification cycles. Libraries were sequenced to a target coverage of approximately 0.1×. Sequencing reads were aligned to the canFam6 genome (GCF_000002285.5) using bwa 0.7.18 and samtools version 1.21 (24). After deduplication with samtools, read depths in 500 kb bins were calculated using bedtools 2.31.1 (25). The cfDNA tumor fraction was inferred using a modified version of ichorCNA version 0.3.2 described previously (19). Long-read sequencing libraries were prepared with 3 ng DNA input using a combination of the KAPA HyperPrep (Roche) and the Native Barcoding kit (Oxford Nanopore Technologies), as described previously (26), with the following modifications: skipping the end repair prior to the adaptor ligation and the use of the adapter from the Native Barcoding kit instead of the AMX-F adapter. Nanopore sequencing reads were aligned to the canFam6 genome using minimap2 version 2.28 (27). Whole-genome libraries for human samples were prepared with the ThruPLEX DNA-Seq HV kit (Takara), and sequencing was performed on the NextSeq 2000 (Illumina), as recently described (medRxiv 2024.12.13.24318579). Human sequencing data were aligned to hgT2T version 2.0 (28) using a similar pipeline as described above for canine sequencing data.

### Classification model development

We used samples from healthy dogs and dogs with cancer to assess classification model performance, using the MLJ.jl framework for random forest implementation. We used a hold-one-dog-out cross-validation approach for random forest training, wherein a model was trained on all samples except those belonging to a test dog, and the model score returned for samples from the test dog was recorded. This was repeated such that each dog’s samples were compared against a model in which they were held out of training, and the ROC plots were generated using the model scores for all samples when they were held out. We performed this analysis twice: once using all samples (so dogs with multiple samples had all their samples held out in each iteration) and once using only pretreatment samples (one per dog, so one sample was held out for each iteration).

### Statistical analysis

Patient groups were compared using nonparametric Mann–Whitney U tests, using the Python package *SciPy* or HypothesisTests.jl in Julia, and plots were prepared using the Makie.jl package.

## Results

### Sample cohorts

A total of 109 canine patients were enrolled in this study through the University of Wisconsin Veterinary Care, including 55 healthy dogs and 54 dogs diagnosed with sarcomas (Table 1). Healthy dogs were confirmed to be cancer-free based on physical exams, blood work, thoracic radiographs, and abdominal ultrasounds as part of the Vaccination Against Canine Cancer Study (29). One healthy dog was excluded from analysis after plasma cfDNA sequencing revealed significant somatic copy-number alterations, suggesting the presence of undiagnosed cancer. The cancer group included dogs with a confirmed diagnosis based on cytology or histopathology. Healthy human samples were collected through the UW BioBank. Canine samples were collected in EDTA or Streck tubes depending on dog size (and collectible blood volume), whereas human samples were collected in EDTA tubes. cfDNA extraction and sequencing library preparation kits were the same for both species (Supplementary Table S1).

**Table 1.** Study samples.

​	UW canine healthy	UW canine cancer	UW human	Commercial canine	Commercial human
Donors	55	54	35	1	1
Samples	55	200	35	1	1
Excluded	1	0	0	0	0
TapeStation	36	76	35	0	0
qPCR and Illumina low-pass WGS	51	194	NA	0	0
qPCR	1	4	NA	0	0
Illumina low-pass WGS	1	1	35	0	0
Nanopore low-pass WGS	2	2	0	1	1

Abbreviation: NA, not applicable; WGS, whole-genome sequencing.

The median age of dogs with cancer was 10.5 years (IQR 3) compared with 10 years (IQR 3) in the healthy cohort (Table 2). The cancer cohort consisted of 26 females and 28 males, whereas the healthy cohort included 31 females and 23 males. In both cohorts, sporting dogs were the most common breed group. Among dogs with cancer, the most represented breeds were Labrador Retriever (*n* = 9), mixed breed (*n* = 8), Great Dane (*n* = 4), and Golden Retriever (*n* = 4). Within the cancer cohort, 32 dogs were diagnosed with osteosarcoma, 13 with hemangiosarcoma, 2 with both osteosarcoma and a second sarcoma, and 7 with other types of high-grade sarcomas (Supplementary Data Table S1). The human sample collection consisted of 27 females and eight males, with a median age of 37 years (range, 22–59 years; and IQR 16.5 years).

**Table 2.** Canine patient cohort characteristics.

​	Cancer cohort (*n* = 54 dogs)	Healthy cohort (*n* = 54 dogs)	Statistical test (*P* value)
Sex	26 females	31 females	Fisher’s exact test (0.09)
Reproductive status	50 spayed or neutered	50 spayed or neutered	—
Age, years (median, IQR)	10.5 (3)	10 (3)	Mann–Whitney U (0.20)
Breed group (American Kennel Club and UK Kennel Club)	16 sporting	21 sporting	—
	13 working	5 working	
	6 hound	1 hound	
	2 terrier	2 terrier	
	5 herding	3 herding	
	1 toy	2 nonsporting	
	11 mixed breeds	20 mixed breeds	
Weight, Kg (median, IQR)	33 (13.2)	28.1 (10.1)	Mann–Whitney U (0.01)
Tumor type	34 OSA	14 benign tumors	—
	13 HSA		
	7 other sarcomas		
Metastasis	13 yes	—	—
​	11 suspected 30 no	​	​

Abbreviations: HSA, hemangiosarcoma; OSA, osteosarcoma; STS, soft-tissue sarcoma.

### Canine cfDNA contain a higher proportion of long DNA fragments compared with human cfDNA

**Figure 1.**
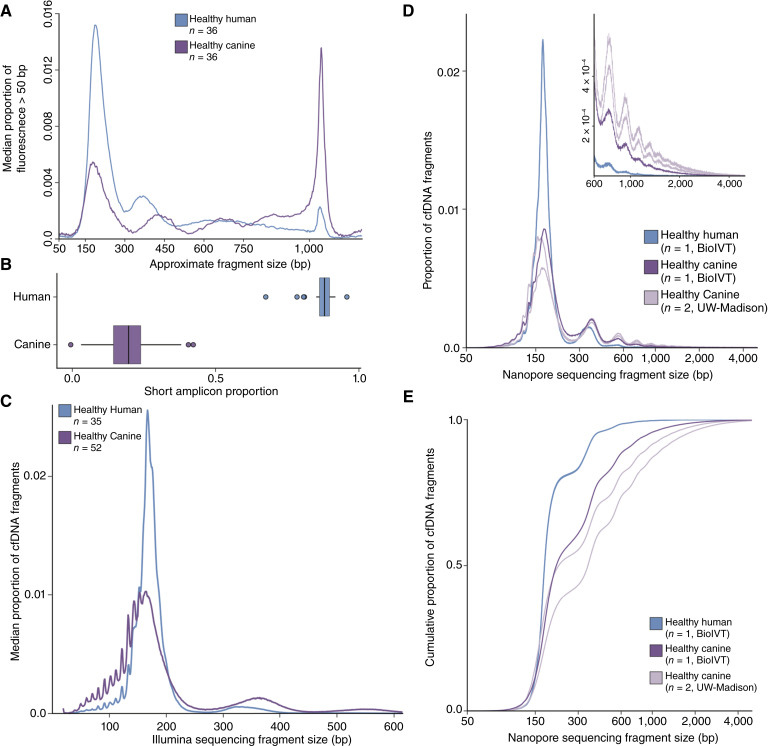
Comparison of cfDNA fragment size distributions in dogs and humans. **A,** Median human and canine cfDNA fragment lengths measured by electrophoresis. Dogs have a significantly higher proportion of longer DNA fragments. **B,** Proportion of short DNA fragments determined by the ratio of amplicons with different lengths in a multiplexed qPCR assay. **C,** Fragment size distributions of human and canine cfDNA derived from short-read sequencing. **D,** Fragment size distributions of human and canine cfDNA derived from long-read cfDNA sequencing. Four samples were analyzed including cfDNA extracted from commercially obtained human (blue) and canine (dark purple) plasma samples, as well as plasma samples from two healthy dogs enrolled at our institution (light purple). The inset shows the 600–5,000 bp range in detail. **E,** Cumulative proportion of fragment sizes from long-read sequencing shown in **D**.

Multimodal analysis of plasma cfDNA revealed a significant proportion of DNA fragments longer than 500 bp, in both healthy dogs and dogs with cancer. When comparing fragment size distributions between humans and healthy dogs using automated electrophoresis, several distinct differences were identified (Fig. 1A). In the 50 to 700 bp range, in which the majority of human cfDNA typically resides, canine cfDNA showed a markedly reduced proportion of mononucleosomal fragments (∼150 bp), along with an increase of approximately 100 bp in the dinucleosome peak position compared with human samples. The proportion of cfDNA within the 50 to 700 bp range was significantly lower (median 0.419 vs. 0.836) and more variable (IQR 0.141 vs. 0.078) in dogs than in humans (*P* < 0.0001). At longer fragment sizes, canine cfDNA exhibited an increase in the proportion of fragments exceeding 800 bp, with the most pronounced difference observed above 1,000 bp.

To evaluate differences in plasma DNA fragmentation using an orthogonal approach, we developed a qPCR assay with amplicons of different sizes. We combined primers amplifying a 598 bp repetitive sequence within the *PECAM1* gene, which occurs 1,997 times in the canFam6 genome, with a previously identified 99 bp sequence within the *L1TD1* gene, a LINE element with 2,634 repeats in the genome (Supplementary Fig. S1; Supplementary Table S2; ref. 22). To establish a quantitative standard curve, we used canine genomic DNA from peripheral blood cells in total amounts ranging from 3 × 10^−5^ ng to 20.2 ng. The assay was validated using buffy coat DNA from 20 healthy dogs (Supplementary Fig. S2; Supplementary Data Table S2). Both LINE and PECAM assays for DNA quantification were strongly correlated with Qubit quantification (R^2^ > 0.95), while slightly overestimating the amount of cfDNA relative to Qubit (linear model intercepts of 0.16 and 0.43, respectively; paired *t* test *P* < 0.001 for each), potentially due to a systematic difference between buffy coat samples tested and the one used for standard curve generation. This could include breed-specific differences in *LINE* or *PECAM1* copies or an underestimated total concentration of the sample used for standard curve generation. Nevertheless, the strong correlation between fluorometry and qPCR quantification indicates that any such differences are minor and permit effective comparison between tested samples.

Using this qPCR-based assay, we calculated the median fraction of short fragments in canine plasma to be 0.196, with an IQR of 0.095. For comparison, our previous work in healthy humans using a similar PCR-based method reported a median fraction of short fragments of 0.88 (IQR 0.035; *P* < 0.0001; Fig. 1B; ref. 23). In addition, the median plasma DNA concentration in healthy dogs measured using qPCR was lower than our earlier results in healthy humans (2.7 ng/mL, IQR 1.13 vs. 6.1 ng/mL, IQR 4.6, *P* < 0.001, Supplementary Data Tables S3–S5).

We next performed Illumina short-read whole-genome sequencing on cfDNA from healthy dogs and humans to assess differences in specific fragment size distributions (Fig. 1C). In canine cfDNA, the mononucleosomal peak is slightly shorter than in humans (153 vs. 166 bp) and shows a greater proportion of fragments below 150 bp while still displaying the characteristic 10 bp periodicity expected due to enzymatic degradation. The broader dinucleosome peak was also evident with a 100 bp size increase in dogs.

Finally, we used Oxford Nanopore long-read sequencing to analyze commercially sourced human and canine plasma cfDNA, as well as two healthy canine samples from our cohort (Fig. 1D and E). In canine cfDNA, the mononucleosome peak was less pronounced, the dinucleosome peak was found at a larger size (Fig. 1D), and there was a notably higher proportion of longer fragments compared with human cfDNA (Fig. 1E). Periodicity in fragment size proportion persisted even beyond 600 bp [Fig. 1D (inset)], suggesting the presence of polynucleosomal fragments in canine cfDNA. Overall, the proportion of cfDNA fragments in the 150 to 250 bp range was lower in dogs compared with humans, whereas the proportion of fragments both below 150 bp and above 250 bp was higher in canine samples.

### Ruling out sample processing artifacts in canine blood collection and processing

In human cfDNA, a high proportion of longer DNA fragments are typically attributed to delays or artifacts during sample processing (23). To rule out this explanation for our observations, we performed additional evaluations of our sample processing protocols (Supplementary Fig. S3). In a subset of 18 dogs, we compared total cfDNA concentration and short fragment index between two consecutively collected blood tubes from each dog. No significant differences were observed in cfDNA concentrations (*P* = 0.38) or the fraction of short fragments (*P* = 0.22) measured by qPCR, suggesting that factors such as additive carryovers or other collection-related variables did not affect our results. We also compared blood samples processed in two independent laboratories; six samples processed in the first lab were compared with 248 samples processed in the second lab. No significant differences were observed in cfDNA concentration (*P* = 0.15) or in the proportion of short DNA fragments (*P* = 0.87). These findings demonstrate that our sample processing protocols were reproducible across collection conditions and laboratories, ruling out processing artifacts as a potential explanation for observed differences between humans and dogs.

### Canine cfDNA fragments in dogs with sarcomas are shorter than in healthy dogs

**Figure 2.**
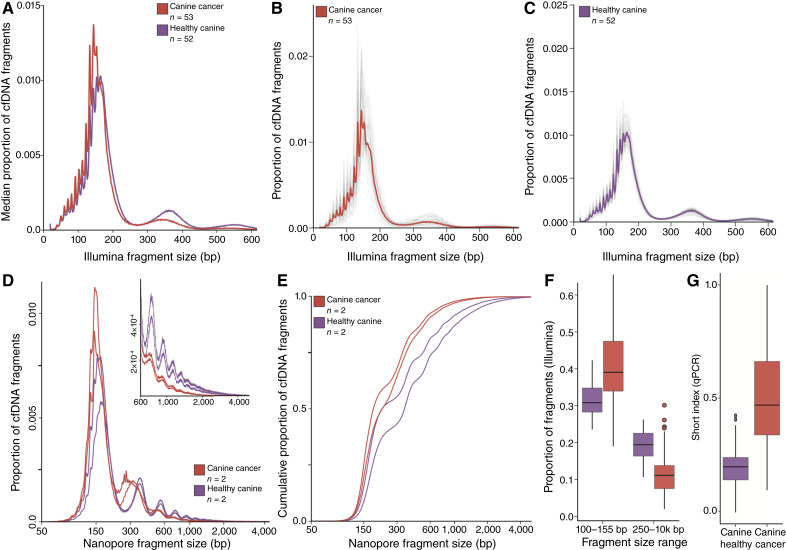
cfDNA fragment size distributions in healthy dogs and dogs with cancer. **A,** Median fragment size distribution derived from short-read sequencing of pretreatment samples from dogs with cancer vs. healthy dogs. Dogs with cancer have shorter cfDNA fragment sizes. **B,** Fragment size distributions for all pretreatment cancer samples (individual gray lines) along with the median shown in **A** (red). **C,** Fragment size distributions for all healthy samples (individual gray lines) along with the median shown in **A** (purple). **D,** Fragment size distributions from long-read sequencing in canine cfDNA samples from dogs with cancer (red) and healthy dogs (purple). The inset shows the 600 to 5,000 bp range in detail. **E,** Cumulative fragment size proportion derived from long-read sequencing data. **F,** Proportion of short read–derived cfDNA fragments in the short (100–155 bp) and long (250 bp+) size ranges, calculated using 52 samples from healthy dogs and 196 samples from dogs with cancer, including pre- and posttreatment samples. **G,** Short index (proportion of short cfDNA fragments) inferred from 51 healthy canine samples and 232 samples from dogs with cancer using qPCR.

We investigated differences in cfDNA fragmentation patterns between dogs with cancer and healthy controls using short-read sequencing, long-read sequencing, and qPCR. A total of 198 plasma samples were analyzed from 54 dogs diagnosed with sarcomas (mean 3.6 samples per dog, range 1–14), including 53 samples collected prior to treatment. We found a shift toward shorter cfDNA fragments in pretreatment plasma samples from dogs with cancer, in both short-read sequencing data (Fig. 2A–C) and long-read sequencing data (Fig. 2D and E), analogous to human cfDNA. In the short-read sequencing data, the modal fragment size decreased from 153 bp in healthy dogs to 144 bp in dogs with cancer. There was also a reduction in the dinucleosome proportion (250–400 bp), along with an increase in the proportion of shorter fragments (50–155 bp), which retained the characteristic 10 bp periodicity observed in human cfDNA. The median fraction of short fragments (100–155 bp) was higher and more variable in dogs with cancer versus healthy dogs (0.42 vs. 0.31; IQR 0.17 vs. 0.07, *P* < 0.0001). In contrast, the median fraction of long fragments (>250 bp) was significantly lower in dogs with cancer (0.121 vs. 0.194, *P* < 0.0001, Fig. 2F). qPCR analysis (Fig. 2G) confirmed these findings, showing a higher median fraction of short fragments in dogs with cancer (0.47 vs. 0.20; IQR 0.33 vs. 0.10; *P* < 0.0001). Dogs with confirmed or suspected metastases had a higher fraction of short fragments than those without metastasis (Supplementary Fig. S4A; median 0.50 vs. 0.42; *P* = 0.003).

To further characterize the cfDNA profiles, we quantified the fraction of cfDNA derived from tumor cells (TF) in all samples using ichorCNA, which infers TF based on deviations in sequencing depth resulting from copy-number aberrations (30). Using a minimum detection threshold of TF > 0.03, tumor-derived cfDNA was detected in 107 of 197 samples (median TF 0.184, IQR 0.288). This is notably higher than the median tumor DNA fraction found in human patients with early-stage cancer (9), consistent with cancer being diagnosed late in dogs. Dogs with suspected or confirmed metastases had a higher TF than those without metastasis (Supplementary Fig. S4B, median 0.10 vs. undetected, *P* = 0.0014). TF was moderately correlated with cfDNA fragment size distributions, showing a positive correlation with the proportion of short fragments (50–150 bp, Spearman rho = 0.64) and a negative correlation with the proportion of fragments in the >250 bp range (Spearman rho = −0.51; Supplementary Fig. S4C and S4D).

### Nucleotide frequencies at fragment ends in canine cfDNA show less bias toward C-ends

**Figure 3.**
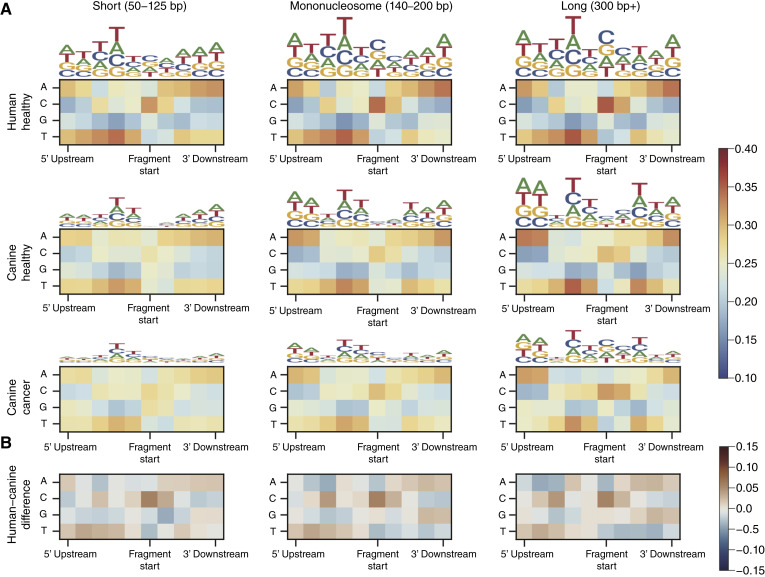
Nucleotide frequencies at fragment ends. **A,** Heatmaps show the median frequencies of each base surrounding cfDNA fragment ends based on Illumina sequencing data. Logo plots visualize the same data, ranking each base with total height based on total bias at the position. Darker colors (red/blue) indicate a stronger preference for or against a given nucleotide at each position. Canine cfDNA is less biased than human cfDNA, especially in dogs with cancer. **B,** The difference in nucleotide bias at fragment ends between healthy human and healthy canine, with red (values above 0) indicating a higher abundance of a given nucleotide/position in human cfDNA and blue indicating a higher abundance in canine cfDNA.

We analyzed the nucleotide composition surrounding cfDNA fragment ends in healthy humans, healthy dogs, and dogs with cancer (Fig. 3). Although human cfDNA shows a strong enrichment of cytosine at the fragment termini, canine cfDNA fragments exhibited minimal nucleotide bias at the two bases immediately downstream of the 5′ fragment end. In both human and canine cfDNA, shorter fragments (50–125 bp) display less pronounced sequence bias compared with longer fragments. Interestingly, canine cfDNA did show an enrichment of thymine in the two bases upstream of the fragment end, a pattern also observed in human cfDNA. Furthermore, samples from dogs with cancer exhibited reduced sequence bias relative to healthy dogs across all fragment size ranges, suggesting that disease state may influence the fragmentation patterns and nuclease activity reflected in cfDNA ends.

### Analysis of canine cfDNA fragmentation metrics enables cancer detection in dogs

**Figure 4.**
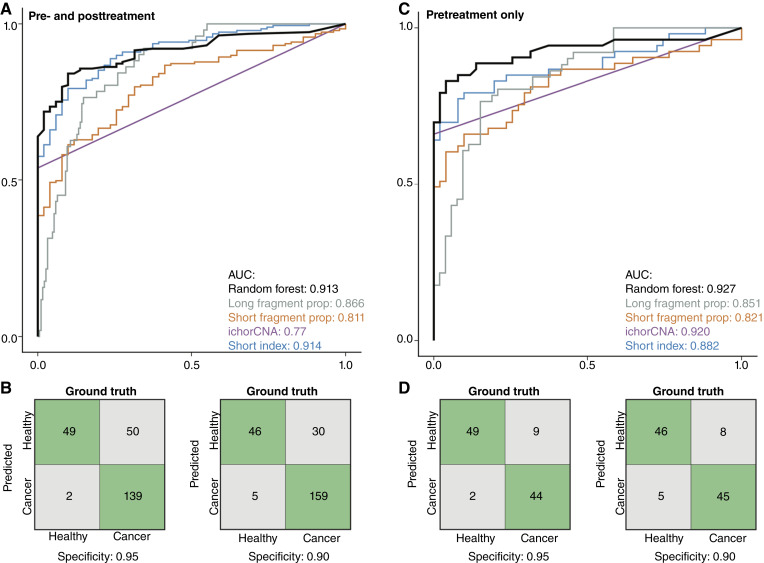
Cancer sample classification performance. ROC plots for the classification of healthy and cancer-derived plasma samples using individual features including short index by qPCR (blue), short fragment proportion by low-pass whole-genome sequencing (LPWGS; orange), long fragment proportion by LPWGS (gray), tumor fraction by ichorCNA (purple), and a random forest combining all features (black). The model was trained using plasma samples from healthy dogs and pretreatment samples from dogs with sarcomas. **A,** Hold-one-dog-out cross-validation performance on all samples, including posttreatment samples. **B,** Confusion matrices at specificities of 0.95 (left) and 0.90 (right) for all samples analyzed, showing the number of correctly classified samples in green and incorrectly classified samples in gray. **C,** Classification performance using hold-one-out cross-validation on pretreatment samples only. **D,** Confusion matrices at specificities of 0.95 (left) and 0.90 (right) for the cross-validation analysis of pretreatment samples only.

cfDNA fragmentation features have become common metrics used to train machine learning classifiers to distinguish healthy individuals from those with cancer. We evaluated the performance of various canine cfDNA fragmentation metrics for distinguishing dogs with sarcomas from healthy controls and developed an exploratory random forest classifier using a leave-one-dog-out cross-validation approach, in which all samples from a single dog were held out of training for validation of each model iteration (Fig. 4). Features assessed included the short fragment index measured by qPCR, tumor fraction measured by ichorCNA, and the proportions of short (100–155 bp) and long (250–10,000 bp) cfDNA fragments derived from whole-genome sequencing. Among the individual features, the short fragment index demonstrated the highest classification performance, achieving an area under the curve (AUC) of 0.914. The proportion of long fragments had an AUC of 0.866 though its sensitivity was limited at high specificity. The random forest classifier using all features achieved an AUC of 0.913, comparable with the short fragment index alone, but demonstrated improved sensitivity at 90% specificity (84.1% for random forest vs. 79.4% for short index, Fig. 4A and B). These results were consistent when the samples used in cross-validation were restricted to the pretreatment blood sample from the held-out dog (Fig. 4C and D). In these samples, incorporating all four features into the random forest model further improved performance, yielding an AUC of 0.927 and a sensitivity of 84.9% at 90% specificity.

## Discussion

The results of this study demonstrate that similar to liquid biopsies in humans, blood-based detection of cancer DNA is feasible in dogs using analysis of plasma DNA fragmentation (16, 17). Our findings highlight the opportunity to perform comparative, observational, and interventional studies for naturally occurring cancers in dogs that can aid biomarker development and improve outcomes for both species. Analysis of plasma DNA has shown promising results for early detection of cancer in humans, but the incidence of cancer in humans spans decades, and the individual risk of developing cancer within a fixed interval of clinical follow-up is often quite low. This necessitates large, expensive cohort studies to evaluate both accuracy and efficacy at improving survival for each newly developed method. Dogs have a compressed lifespan but a cancer incidence similar to that of humans, making early detection studies more feasible and cost-effective. Prospective studies of early cancer detection in companion dogs are also distinct from laboratory animal models because cancers in dogs occur spontaneously and allow repeated sample collection throughout the disease course.

We found that plasma DNA fragmentation profiles were distinct in dogs, different from those observed in both humans and mice (31). Although the expected fraction of cfDNA less than 700 bp in length is greater than 80% in human plasma samples, none of the plasma samples from healthy dogs showed a fraction greater than 50%. We found that the fragment length distribution of plasma DNA in dogs shows a mononucleosomal peak at lower relative abundance, as seen earlier (18), and ∼10 bp shorter than the expected fragment length of ∼167 bp. In contrast, the dinucleosomal peak in dogs was ∼100 bp longer than the corresponding peak in humans.

Although our findings were consistent across multiple fragmentation assays and nearly 250 plasma samples collected from more than 100 dogs, it is unclear what biological differences drive this observation. In humans, plasma cfDNA is thought to be largely of apoptotic origin. Chromatin digestion into mononucleosomes is driven by intracellular DNA fragmentation factor B followed by DNase1-L3 and DNase1 in plasma (31). In dogs, we speculate that there may be differences in the activity or concentration of circulating nucleases, or differences in chromatin accessibility, that drive the observed fragment lengths in plasma DNA. It is also possible that nonapoptotic processes contribute a large fraction of cfDNA in dog plasma, such as DNA associated with neutrophil extracellular traps. Although the mechanisms behind these differences will require further study, characterization of these differences in plasma DNA fragmentation between dogs and humans can guide the development and evaluation of new plasma DNA assays in both species.

We found no difference in results obtained from multiple paired blood tubes or plasma obtained from different processing sites or commercial vendors. If the increased contribution of longer DNA fragments were due to blood cell lysis during sample processing, we would also expect an increase in the total concentration of DNA. On the contrary, we found that plasma DNA concentration in dogs was lower than in humans on average. The fragment size distributions we found in the current healthy cohort of 54 dogs were slightly smaller than those found in nine healthy dogs analyzed in our previous work (modal size 153 bp in this study cohort vs. 165 bp in Favaro and colleagues 2022, Supplemental Fig. S5). The overall size distributions look very similar but are shifted by about 10 bp, a difference likely driven by additional size selection as a result of FAB treatment during library preparation in the earlier study to address sample index hopping issues with the Illumina patterned flow cell chemistry at the time.

Our study is limited by a relatively small sample size of ∼250 samples from ∼100 dogs, and our samples from dogs with cancer are limited to multiple types of sarcoma. This prevents precise assessment of effect size or assessment of variation in cfDNA features across cancer types. In addition, classification was performed between dogs who were diagnosed with cancer under the current standard of care and cancer-free dogs. Future studies incorporating larger sample sizes across diverse histologies, particularly highly prevalent cancers such as lymphoma, will be essential to establish the broader clinical applicability of this methodology.

In summary, our findings highlight differences in fragmentation between humans and dogs and suggest that plasma DNA analysis using qPCR and low-depth whole-genome sequencing could enable cancer detection and treatment monitoring in dogs, both in veterinary practice and in comparative oncology studies. Future studies are warranted to validate these findings in a larger, prospective cohort across multiple cancer types and to evaluate the diagnostic performance of fragmentation analysis for cancer detection in dogs. In addition, the differences in plasma DNA fragmentation patterns observed between dogs and humans warrant further investigation into the physiologic mechanisms involved in cfDNA generation and degradation.

## Supplementary Material

Table S1 Table S1. Human and canine sample collection comparison.

Table S2 Table S2. Primers and probes utilized for the real-time quantitative PCR (qPCR) targeting segments of the canine genome.

Figure S1 Schematic representation of sequence fragments corresponding to the primers LINE99 and PECAM598 across the canine genome (canFam6). A. The LINE99 assay. B. The PECAM598 assay.

Figure S2 Supplementary Figure S2. Validation of a qPCR-based DNA quantification method for short and long cfDNA. A. Comparison of quantification of fragments using the LINE primers with quantification using fluorometry (Qubit) in buffy coat samples. B. Comparison of quantification of fragments using the PECAM primers versus quantification using fluorometry (Qubit) in buffy coat samples. C. Comparison of cfDNA concentration per ml of plasma in human and canine healthy cfDNA samples.

Figure S3 Evaluation of pre-analytical variables in canine cfDNA collection and processing. A. Comparison of DNA concentration by qPCR (LINE99) between the first and second blood tubes collected from each dog (n=18, paired p = 0.38). B. Comparison of fraction of short fragments measured by qPCR (short index) between the first and second blood tubes collected from each dog (n=18 pairs, paired p = 0.22). C. Comparison of DNA concentration by qPCR (LINE99) between samples processed by two different operators in two different lab environments (n=6 for laboratory A and n=281 for laboratory B, unpaired p = 0.12) D. Comparison of fraction of short fragments measure by qPCR (short index) between samples processed by two different operators in two different lab environments (n=6 for laboratory A and n=281 for laboratory B, unpaired p = 0.98).

Figure S4 A. Distribution of short index values for plasma samples taken from dogs with suspected or confirmed metastases, or without them. B. Tumor fraction estimated using ichorCNA for dogs with or without suspected/confirmed metastases. C. Correlation between the proportion of cfDNA fragments in the range 100-155bp and ichorCNA tumor fraction. D. Correlation between the proportion of cfDNA fragments in the range 250-10000bp and ichorCNA tumor fraction.

Figure S5 Comparison of canine cfDNA fragment size distributions found in this study (modal size 153bp, n=54) versus our previous work (modal size 165bp, n=9, Favaro et al 2022), along with the human cfDNA fragment size distribution (modal size 166bp, n =35).

## Data Availability

The canine sequencing data generated in this study are available at NCBI Sequence Read Archive (PRJNA1373368). Code and data tables needed to reproduce the figures and analysis are available on Zenodo (https://dx.doi.org/10.5281/zenodo.17555907). All other data are available from the corresponding author upon request.
