# Supplementary material for: Comparative Analysis of Cell-Free DNA Fragmentation Patterns in Canines with Sarcoma and Tumor-Free Canines and Humans
Source: Cancer Res Commun. 2026 Feb 13;6(2):310–9. doi: 10.1158/2767-9764.CRC-25-0373 (PMC13037772; doi:10.1158/2767-9764.CRC-25-0373)
Supplement: Table S1 — Human and canine sample collection comparison. [file crc-25-0373_table_s1_suppst1.docx]

|  | UW human-healthy | UW canine-cancer | UW canine-healthy |
| --- | --- | --- | --- |
| Number of individuals | 35 | 54 | 54 |
| Number of samples | 35 | 200 | 54 |
| Tube type | K2 EDTA | Streck (n=194)  K2 EDTA (n=6) | Streck |
| Extraction kit | MagMAX cfDNA | MagMAX cfDNA | MagMAX cfDNA |
| Extraction volume | 1mL | 2-3mL | 2-3mL |
| Library preparation kit | ThruPLEX Plasma-seq | ThruPLEX Plasma-seq | ThruPLEX Plasma-seq |
| Sequencer | Illumina NextSeq 2000 | Illumina NextSeq 2000 | Illumina NextSeq 2000 |
| Reference genome | hgT2T | canFam6 | canFam6 |
