# Supplementary material for: Comparative Analysis of Cell-Free DNA Fragmentation Patterns in Canines with Sarcoma and Tumor-Free Canines and Humans
Source: Cancer Res Commun. 2026 Feb 13;6(2):310–9. doi: 10.1158/2767-9764.CRC-25-0373 (PMC13037772; doi:10.1158/2767-9764.CRC-25-0373)
Supplement: Table S2 — Primers and probes utilized for the real-time quantitative PCR (qPCR) targeting segments of the canine genome. [file crc-25-0373_table_s2_suppst2.docx]

| Oligo ID | Sequence 5' to 3' | Probe label |
| --- | --- | --- |
| LINE1can99_Forward | AAATGCAATGAAACGCCGGG | FAM |
| LINE1can99_Reverse | TCTTTCGTTGGACACCGAGG |  |
| LINE1can99_Probe | TGCACCCCGATGTTTCTAGCAGCA |  |
| PECAM1can_Forward | CCACAAATGTTGGAGAGGAT | Cy5 |
| PECAM1can_Reverse | CTYATGTTCTGTCTCCCTTTC |  |
| PECAM1can_Probe | ACCTGCCCTATGACCCAGCAATT |  |
