# Supplementary figures and images for: Comparative Analysis of Cell-Free DNA Fragmentation Patterns in Canines with Sarcoma and Tumor-Free Canines and Humans
Source: Cancer Res Commun. 2026 Feb 13;6(2):310–9. doi: 10.1158/2767-9764.CRC-25-0373 (PMC13037772; doi:10.1158/2767-9764.CRC-25-0373)

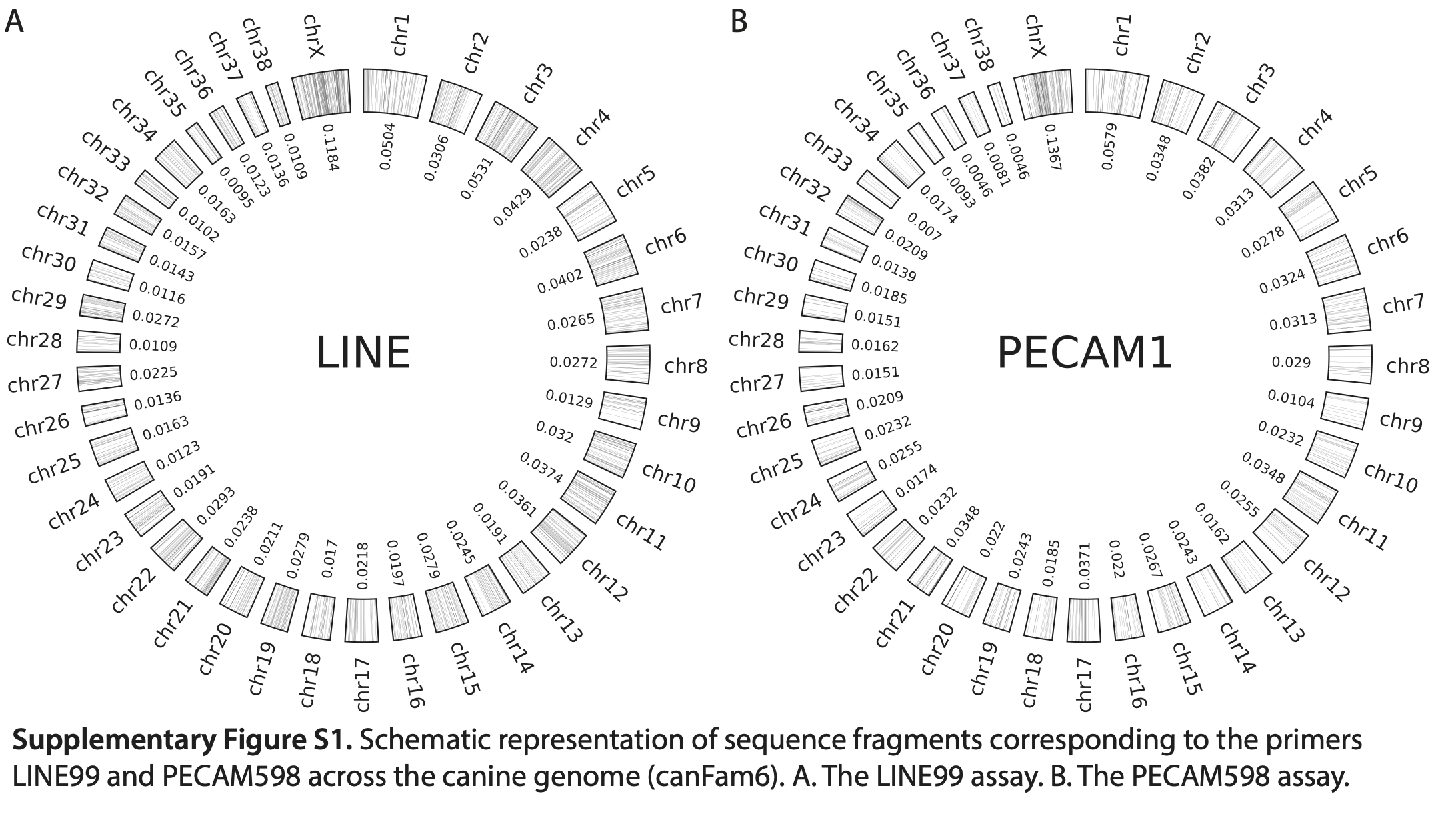

Supplement: Figure S1 — Schematic representation of sequence fragments corresponding to the primers LINE99 and PECAM598 across the canine genome (canFam6). A. The LINE99 assay. B. The PECAM598 assay. [file crc-25-0373_figure_s1_suppsf1.png]

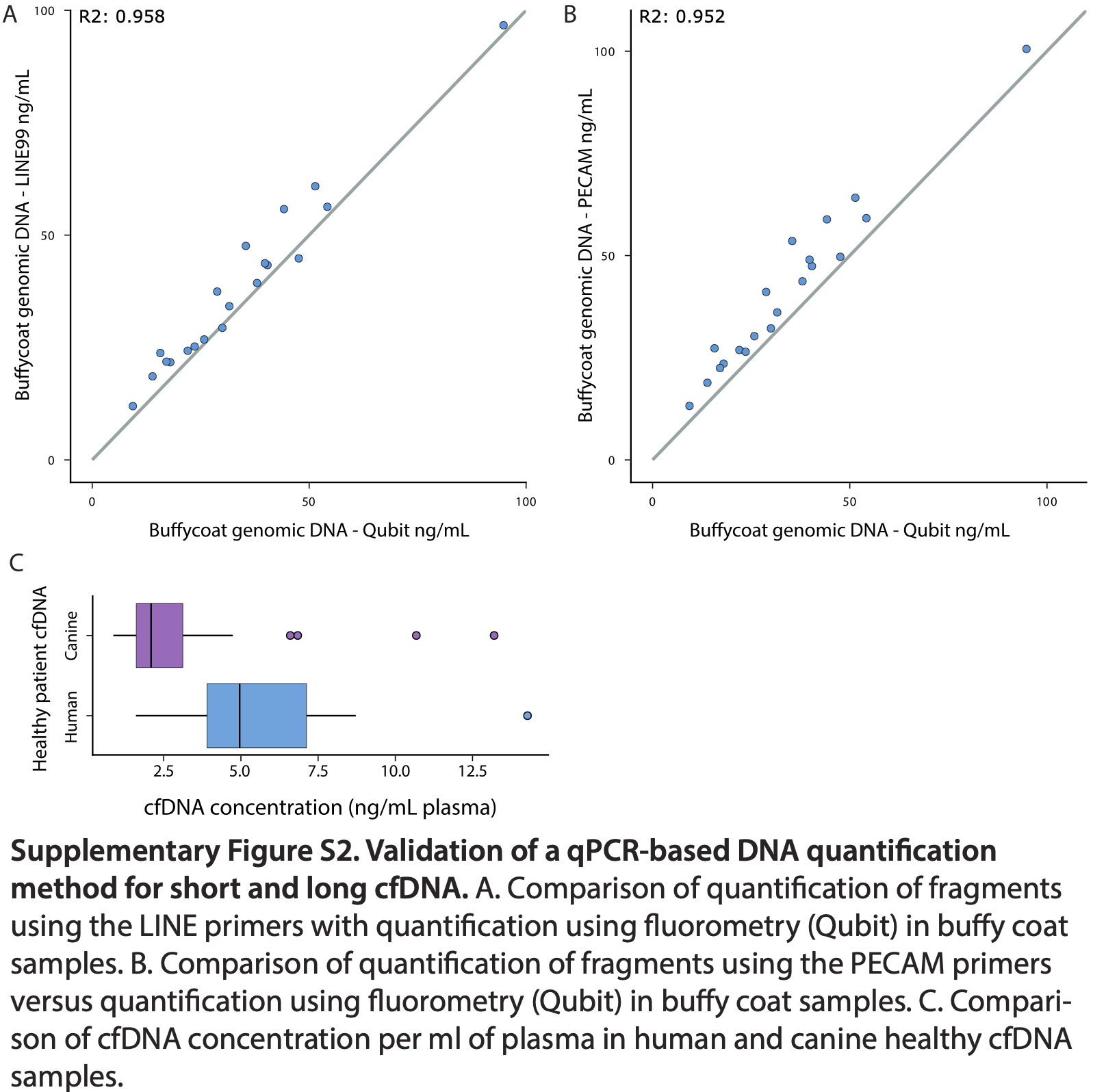

Supplement: Figure S2 — Supplementary Figure S2. Validation of a qPCR-based DNA quantification method for short and long cfDNA. A. Comparison of quantification of fragments using the LINE primers with quantification using fluorometry (Qubit) in buffy coat samples. B. Comparison of quantification of fragments using the PECAM primers versus quantification using fluorometry (Qubit) in buffy coat samples. C. Comparison of cfDNA concentration per ml of plasma in human and canine healthy cfDNA samples. [file crc-25-0373_figure_s2_suppsf2.png]

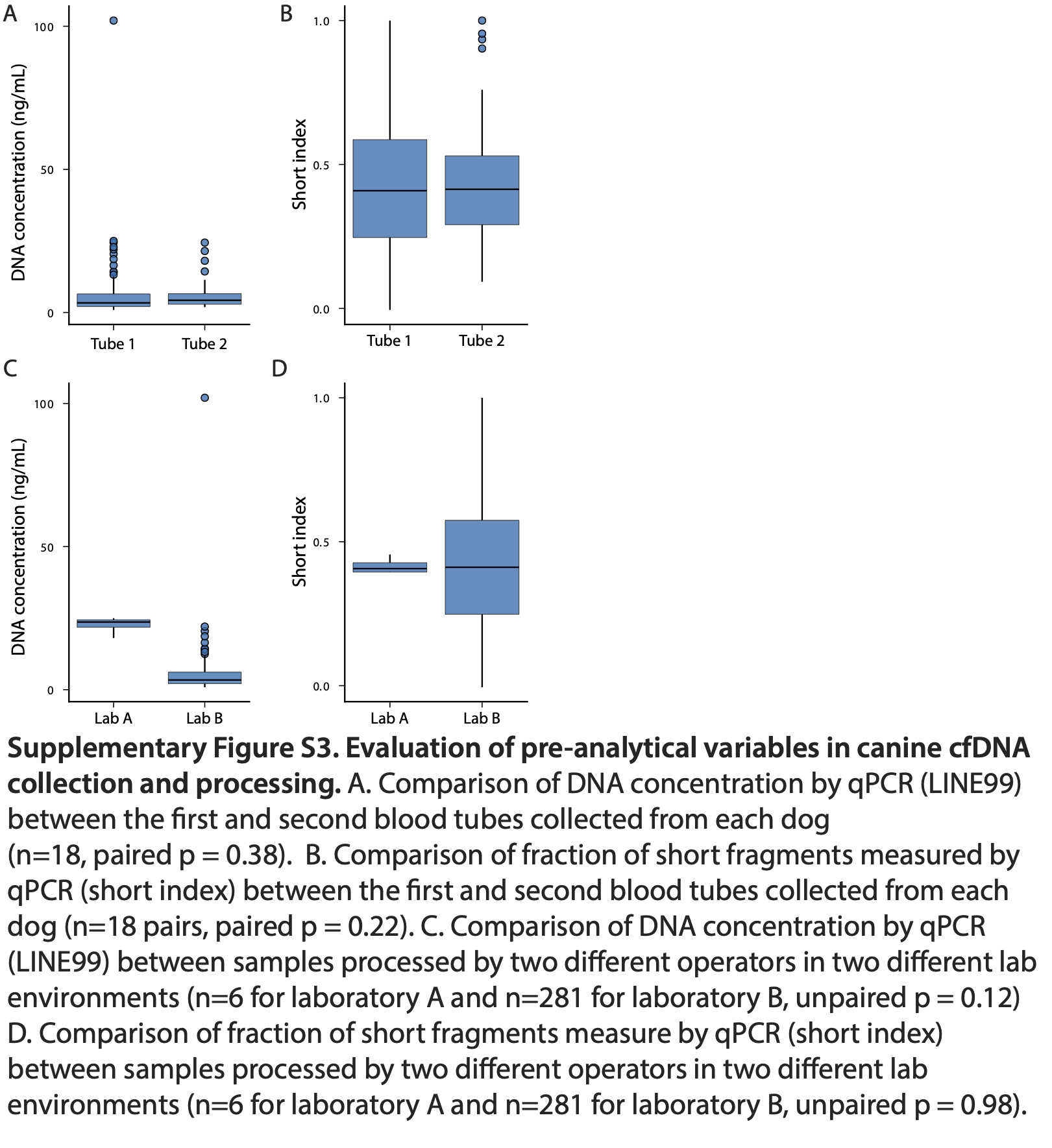

Supplement: Figure S3 — Evaluation of pre-analytical variables in canine cfDNA collection and processing. A. Comparison of DNA concentration by qPCR (LINE99) between the first and second blood tubes collected from each dog (n=18, paired p = 0.38). B. Comparison of fraction of short fragments measured by qPCR (short index) between the first and second blood tubes collected from each dog (n=18 pairs, paired p = 0.22). C. Comparison of DNA concentration by qPCR (LINE99) between samples processed by two different operators in two different lab environments (n=6 for laboratory A and n=281 for laboratory B, unpaired p = 0.12) D. Comparison of fraction of short fragments measure by qPCR (short index) between samples processed by two different operators in two different lab environments (n=6 for laboratory A and n=281 for laboratory B, unpaired p = 0.98). [file crc-25-0373_figure_s3_suppsf3.png]

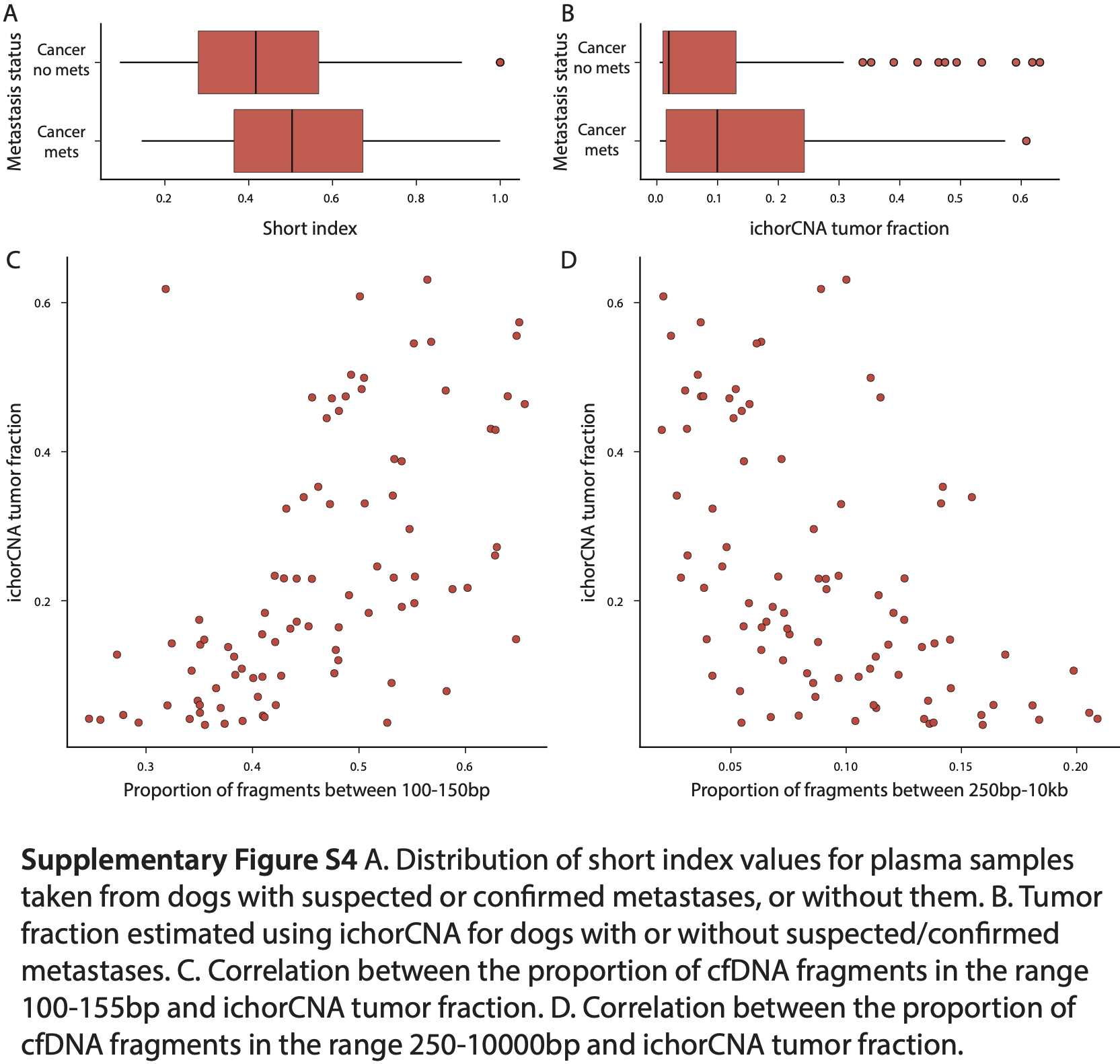

Supplement: Figure S4 — A. Distribution of short index values for plasma samples taken from dogs with suspected or confirmed metastases, or without them. B. Tumor fraction estimated using ichorCNA for dogs with or without suspected/confirmed metastases. C. Correlation between the proportion of cfDNA fragments in the range 100-155bp and ichorCNA tumor fraction. D. Correlation between the proportion of cfDNA fragments in the range 250-10000bp and ichorCNA tumor fraction. [file crc-25-0373_figure_s4_suppsf4.png]

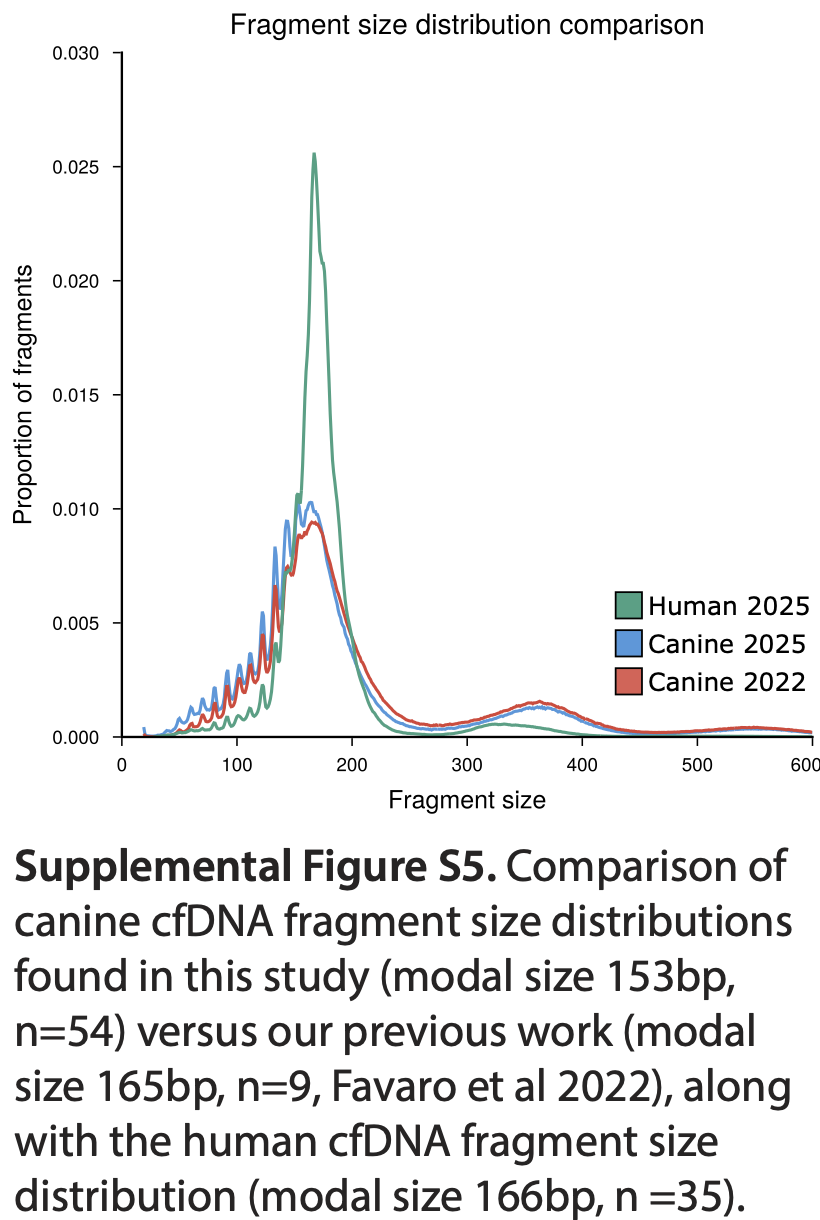

Supplement: Figure S5 — Comparison of canine cfDNA fragment size distributions found in this study (modal size 153bp, n=54) versus our previous work (modal size 165bp, n=9, Favaro et al 2022), along with the human cfDNA fragment size distribution (modal size 166bp, n =35). [file crc-25-0373_figure_s5_suppsf5.png]
